# 4,4′-{[4-(2,2′:6′,2′′-Terpyridin-4′-yl)phen­yl]imino}­dibenzaldehyde

**DOI:** 10.1107/S1600536812014833

**Published:** 2012-04-13

**Authors:** Wei-Biao Shen, Zhi-Wen Zhang, Li-Wen Wang, Jie-Ying Wu

**Affiliations:** a Department of Chemistry, Anhui University, Hefei 230039, People’s Republic of China and, Key Laboratory of Functional Inorganic Materials, Chemistry, Hefei 230039, Peoples Republic of China

## Abstract

The central pyridine ring of the 2,2′:6′,2′′-terpyridine fragment of the title compound, C_35_H_24_N_4_O_2_, forms dihedral angles of 8.3 (2), 10.6 (3) and 39.4 (3)°, respectively, with the two outer pyridine rings and the attached benzene ring. In the crystal, weak C—H⋯O inter­actions link the mol­ecules into chains in [010].

## Related literature

For supra­molecular assemblies and composite fluorescent sensors of related substituted terpyridines, see: Cargill Thompson (1997 ▶); Goodall *et al.* (2002 ▶); Mutai *et al.* (2001 ▶). For related reviews, see: Heller & Schubert (2003 ▶); Fallahpour *et al.* (2003 ▶). For details of the synthesis, see: Krohnke (1976 ▶).
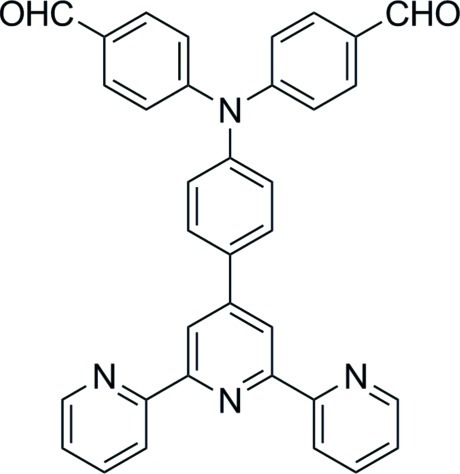


## Experimental

### 

#### Crystal data

C_35_H_24_N_4_O_2_
*M* *_r_* = 532.58
Orthorhombic, 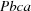

*a* = 11.2518 (14) Å
*b* = 18.380 (2) Å
*c* = 25.860 (3) Å
*V* = 5348.1 (12) Å^3^
*Z* = 8
Mo *K*α radiation
μ = 0.08 mm^−1^
*T* = 296 K
0.20 × 0.10 × 0.10 mm

#### Data collection

Bruker SMART CCD area-detector diffractometer
Absorption correction: multi-scan (*SADABS*; Sheldrick, 1996 ▶) *T*_min_ = 0.983, *T*_max_ = 0.992
36182 measured reflections
4712 independent reflections
3293 reflections with *I* > 2σ(*I*)
*R*_int_ = 0.037

#### Refinement

*R*[*F* ^2^ > 2σ(*F* ^2^)] = 0.042
*wR*(*F* ^2^) = 0.123
*S* = 1.06
4712 reflections
370 parameters
H-atom parameters constrained
Δρ_max_ = 0.23 e Å^−3^
Δρ_min_ = −0.19 e Å^−3^

### 

Data collection: *SMART* (Bruker, 2007 ▶); cell refinement: *SAINT* (Bruker, 2007 ▶); data reduction: *SAINT*; program(s) used to solve structure: *SHELXS97* (Sheldrick, 2008 ▶); program(s) used to refine structure: *SHELXL97* (Sheldrick, 2008 ▶); molecular graphics: *SHELXTL* (Sheldrick, 2008 ▶); software used to prepare material for publication: *SHELXTL*.

## Supplementary Material

Crystal structure: contains datablock(s) I, global. DOI: 10.1107/S1600536812014833/cv5273sup1.cif

Structure factors: contains datablock(s) I. DOI: 10.1107/S1600536812014833/cv5273Isup2.hkl

Supplementary material file. DOI: 10.1107/S1600536812014833/cv5273Isup3.cml

Additional supplementary materials:  crystallographic information; 3D view; checkCIF report

## Figures and Tables

**Table 1:** Hydrogen-bond geometry (Å, °)

*D*—H⋯*A*	*D*—H	H⋯*A*	*D*⋯*A*	*D*—H⋯*A*
C27—H27⋯O1^i^	0.93	2.46	3.339 (3)	158

Symmetry code: (i) 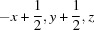.

**Fig. 1.**
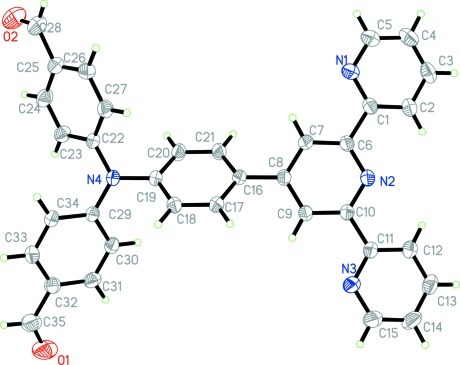
: The molecular structure of (I) showing 30% probability displacement ellipsoids.

## supplementary crystallographic information

### Comment

Substituted terpyridines are frequently used as building blocks for supramolecular assemblies and composite fluorescent sensors (Cargill Thompson, 1997; Goodall *et al.*, 2002; Mutai *et al.*, 2001), and several reviews were published on the subject (Heller *et al.*, 2003; Fallahpour *et al.*, 2003). The title compound (I) can be used as intermediate in the synthesis of terpyridines, and here we report its crystal structure.

In (I) (Fig.1), the central pyridine ring of the 2,2':6',2''-terpyridine fragment forms dihedral angles of 8.3 (2), 10.6 (3) and 39.4 (3) °, respectively, with the two outer pyridine rings and the attached benzene ring. Weak intermolecular C—H···O interactions (Table 1) link the molecules into chains in [010].

### Experimental

DMF (2 g, 27.4 mmol) was added to a three-necked flask in ice equipped with a magnetic stirrer and a reflux condenser, then POCl_3_ (4.0 g, 26.4 mmol) was added dropwisely (about 30 min), then 4'-(4-(diphenylamino)phenyl)-2,2':6',2''-terpyridine)(3.0 g, 6.2 mmol), which was synthesized using Krohnke's method (Krohnke, 1976.), dissolved in 50 ml of chloroform and added to the frozen salt, stirring for 36 h at 355 K. After being cooled to room temperature, the mixture was poured into a large amount of ice water and adjusted to the pH= 8 with sodium hydroxide. After extraction with CH_2_Cl_2_ (4 *x* 20 ml), the combined organic phase was dried over anhydrous Na_2_SO_4_ and evaporated to give a yellow solid. Purification by column chromatography (silica, petroleum: ethyl acetate=5:1, yield: 50%) ^1^H NMR (DMSO-d6, 400 MHz): 9.92 (s, 2H), 8.75–8.77 (m, 4H), 8.69 (d, 2H, J=7.6 Hz), 8.02–8.05 (m, 4H), 7.90 (d, 4H, J=7.6 Hz), 7.53 (t, 2H, J=6.4 Hz), 7.38 (d, 2H, J=8.8 Hz), 7.28 (d, 4H, J=8.4 Hz). MS: m/*z* (%) = 504.20 (100). FT—IR (KBr, cm^-1^): 3464, 3060, 2919, 2850, 1714, 1546, 1508, 1474, 1434, 1365, 1321, 1179, 1111, 1069, 1015, 968, 834, 793, 769, 731, 702, 659, 637, 528.

### Refinement

All hydrogen atoms were placed in geometrically idealized positions and constrained to ride on their parent atoms, with C—H = 0.93 Å and *U*_iso_(H) = 1.2 *U*_eq_(C).

### Crystal data

**Table:**

C_35_H_24_N_4_O_2_	*F*(000) = 2224
*M**_r_* = 532.58	*D*_x_ = 1.323 Mg m^−^^3^
Orthorhombic, *P**b**c**a*	Mo *K*α radiation, λ = 0.71073 Å
Hall symbol: -P 2ac 2ab	Cell parameters from 6054 reflections
*a* = 11.2518 (14) Å	θ = 2.3–23.1°
*b* = 18.380 (2) Å	µ = 0.08 mm^−^^1^
*c* = 25.860 (3) Å	*T* = 296 K
*V* = 5348.1 (12) Å^3^	Block, yellow
*Z* = 8	0.20 × 0.10 × 0.10 mm

### Data collection

**Table:**

Bruker SMART CCD area-detector diffractometer	4712 independent reflections
Radiation source: fine-focus sealed tube	3293 reflections with *I* > 2σ(*I*)
Graphite monochromator	*R*_int_ = 0.037
φ and ω scans	θ_max_ = 25.0°, θ_min_ = 1.6°
Absorption correction: multi-scan (*SADABS*; Sheldrick, 1996)	*h* = −13→13
*T*_min_ = 0.983, *T*_max_ = 0.992	*k* = −21→21
36182 measured reflections	*l* = −30→29

### Refinement

**Table:**

Refinement on *F*^2^	Primary atom site location: structure-invariant direct methods
Least-squares matrix: full	Secondary atom site location: difference Fourier map
*R*[*F*^2^ > 2σ(*F*^2^)] = 0.042	Hydrogen site location: inferred from neighbouring sites
*wR*(*F*^2^) = 0.123	H-atom parameters constrained
*S* = 1.06	*w* = 1/[σ^2^(*F*_o_^2^) + (0.054*P*)^2^ + 1.1184*P*] where *P* = (*F*_o_^2^ + 2*F*_c_^2^)/3
4712 reflections	(Δ/σ)_max_ = 0.001
370 parameters	Δρ_max_ = 0.23 e Å^−^^3^
0 restraints	Δρ_min_ = −0.19 e Å^−^^3^

### Special details

**Table:**

Geometry. All e.s.d.'s (except the e.s.d. in the dihedral angle between two l.s. planes) are estimated using the full covariance matrix. The cell e.s.d.'s are taken into account individually in the estimation of e.s.d.'s in distances, angles and torsion angles; correlations between e.s.d.'s in cell parameters are only used when they are defined by crystal symmetry. An approximate (isotropic) treatment of cell e.s.d.'s is used for estimating e.s.d.'s involving l.s. planes.
Refinement. Refinement of *F*^2^ against ALL reflections. The weighted *R*-factor *wR* and goodness of fit *S* are based on *F*^2^, conventional *R*-factors *R* are based on *F*, with *F* set to zero for negative *F*^2^. The threshold expression of *F*^2^ > σ(*F*^2^) is used only for calculating *R*-factors(gt) *etc*. and is not relevant to the choice of reflections for refinement. *R*-factors based on *F*^2^ are statistically about twice as large as those based on *F*, and *R*- factors based on ALL data will be even larger.

### Fractional atomic coordinates and isotropic or equivalent isotropic displacement parameters (Å^2^)

**Table:**

	*x*	*y*	*z*	*U*_iso_*/*U*_eq_	
C1	0.34362 (17)	1.12523 (9)	0.52197 (7)	0.0503 (5)	
C2	0.40956 (19)	1.17424 (10)	0.49311 (8)	0.0609 (5)	
H2	0.4919	1.1706	0.4917	0.073*	
C3	0.3512 (2)	1.22853 (11)	0.46644 (9)	0.0737 (6)	
H3	0.3937	1.2616	0.4465	0.088*	
C4	0.2298 (2)	1.23319 (12)	0.46969 (10)	0.0818 (7)	
H4	0.1882	1.2694	0.4522	0.098*	
C5	0.1719 (2)	1.18318 (13)	0.49930 (11)	0.0870 (8)	
H5	0.0896	1.1867	0.5016	0.104*	
C6	0.40117 (15)	1.06344 (9)	0.54985 (6)	0.0451 (4)	
C7	0.33297 (15)	1.00924 (9)	0.57239 (7)	0.0488 (4)	
H7	0.2505	1.0116	0.5707	0.059*	
C8	0.38747 (15)	0.95134 (9)	0.59748 (7)	0.0453 (4)	
C9	0.51034 (15)	0.95181 (9)	0.59970 (6)	0.0463 (4)	
H9	0.5504	0.9149	0.6171	0.056*	
C10	0.57403 (15)	1.00742 (9)	0.57600 (6)	0.0436 (4)	
C11	0.70587 (15)	1.00773 (9)	0.57624 (7)	0.0479 (4)	
C12	0.76956 (17)	1.05690 (11)	0.54670 (8)	0.0587 (5)	
H12	0.7300	1.0910	0.5264	0.070*	
C13	0.89120 (19)	1.05502 (13)	0.54752 (9)	0.0708 (6)	
H13	0.9352	1.0875	0.5277	0.085*	
C14	0.94687 (18)	1.00474 (13)	0.57796 (9)	0.0767 (7)	
H14	1.0294	1.0023	0.5793	0.092*	
C15	0.87852 (19)	0.95816 (14)	0.60630 (10)	0.0831 (7)	
H15	0.9169	0.9240	0.6269	0.100*	
C16	0.31729 (15)	0.88945 (9)	0.61836 (7)	0.0457 (4)	
C17	0.34900 (15)	0.85442 (10)	0.66384 (7)	0.0499 (5)	
H17	0.4140	0.8711	0.6826	0.060*	
C18	0.28553 (15)	0.79505 (10)	0.68166 (7)	0.0514 (5)	
H18	0.3082	0.7723	0.7122	0.062*	
C19	0.18861 (15)	0.76917 (9)	0.65435 (7)	0.0470 (4)	
C20	0.15636 (16)	0.80334 (11)	0.60891 (7)	0.0568 (5)	
H20	0.0920	0.7861	0.5899	0.068*	
C21	0.21940 (16)	0.86300 (10)	0.59159 (7)	0.0556 (5)	
H21	0.1957	0.8860	0.5613	0.067*	
C22	−0.00413 (15)	0.71474 (9)	0.67351 (6)	0.0451 (4)	
C23	−0.07646 (16)	0.65568 (10)	0.66192 (7)	0.0542 (5)	
H23	−0.0426	0.6107	0.6546	0.065*	
C24	−0.19783 (17)	0.66359 (12)	0.66123 (8)	0.0629 (5)	
H24	−0.2454	0.6235	0.6541	0.075*	
C25	−0.25059 (18)	0.73015 (12)	0.67098 (8)	0.0620 (5)	
C26	−0.17818 (18)	0.78851 (11)	0.68230 (8)	0.0644 (6)	
H26	−0.2124	0.8337	0.6887	0.077*	
C27	−0.05665 (17)	0.78156 (10)	0.68434 (7)	0.0563 (5)	
H27	−0.0097	0.8214	0.6929	0.068*	
C28	−0.3793 (2)	0.74042 (18)	0.66989 (12)	0.0966 (9)	
H28	−0.4093	0.7851	0.6806	0.116*	
C29	0.17967 (15)	0.64554 (9)	0.69077 (7)	0.0459 (4)	
C30	0.28969 (15)	0.62576 (10)	0.67059 (8)	0.0545 (5)	
H30	0.3239	0.6534	0.6444	0.065*	
C31	0.34788 (17)	0.56553 (10)	0.68920 (8)	0.0580 (5)	
H31	0.4217	0.5531	0.6757	0.070*	
C32	0.29803 (17)	0.52300 (10)	0.72789 (9)	0.0591 (5)	
C33	0.18863 (18)	0.54246 (11)	0.74709 (9)	0.0665 (6)	
H33	0.1538	0.5139	0.7726	0.080*	
C34	0.12955 (16)	0.60295 (10)	0.72950 (8)	0.0559 (5)	
H34	0.0561	0.6154	0.7435	0.067*	
C35	0.3592 (2)	0.45935 (13)	0.74879 (12)	0.0892 (8)	
H35	0.3214	0.4347	0.7756	0.107*	
N1	0.22517 (15)	1.12938 (9)	0.52524 (7)	0.0720 (5)	
N2	0.52047 (13)	1.06278 (7)	0.55086 (5)	0.0467 (4)	
N3	0.75941 (15)	0.95862 (10)	0.60629 (7)	0.0697 (5)	
N4	0.12091 (12)	0.70849 (8)	0.67281 (6)	0.0514 (4)	
O1	0.45218 (17)	0.43587 (9)	0.73507 (9)	0.1106 (7)	
O2	−0.44856 (17)	0.69503 (14)	0.65606 (10)	0.1367 (9)	

### Atomic displacement parameters (Å^2^)

**Table:**

	*U*^11^	*U*^22^	*U*^33^	*U*^12^	*U*^13^	*U*^23^
C1	0.0571 (12)	0.0409 (10)	0.0530 (11)	0.0006 (9)	0.0039 (9)	0.0025 (8)
C2	0.0663 (13)	0.0489 (11)	0.0675 (13)	−0.0052 (10)	0.0007 (10)	0.0078 (10)
C3	0.0953 (18)	0.0521 (13)	0.0735 (14)	−0.0074 (12)	−0.0005 (13)	0.0187 (11)
C4	0.0900 (18)	0.0591 (14)	0.0962 (17)	0.0123 (13)	−0.0114 (14)	0.0217 (13)
C5	0.0669 (15)	0.0736 (15)	0.121 (2)	0.0161 (12)	0.0007 (14)	0.0341 (15)
C6	0.0475 (10)	0.0395 (10)	0.0482 (10)	−0.0007 (8)	0.0057 (8)	0.0008 (8)
C7	0.0398 (10)	0.0467 (10)	0.0597 (11)	0.0015 (8)	0.0058 (8)	0.0025 (9)
C8	0.0451 (10)	0.0411 (10)	0.0496 (10)	−0.0026 (8)	0.0054 (8)	0.0016 (8)
C9	0.0458 (10)	0.0410 (10)	0.0520 (11)	−0.0011 (8)	0.0040 (8)	0.0043 (8)
C10	0.0448 (10)	0.0411 (9)	0.0451 (10)	−0.0026 (8)	0.0035 (8)	−0.0013 (8)
C11	0.0460 (10)	0.0470 (10)	0.0506 (10)	−0.0055 (8)	0.0035 (8)	0.0002 (9)
C12	0.0519 (12)	0.0587 (12)	0.0656 (12)	−0.0069 (9)	0.0079 (9)	0.0106 (10)
C13	0.0561 (13)	0.0763 (15)	0.0800 (15)	−0.0179 (11)	0.0131 (11)	0.0092 (12)
C14	0.0415 (11)	0.1014 (18)	0.0873 (16)	−0.0072 (12)	0.0038 (11)	0.0053 (15)
C15	0.0515 (13)	0.0987 (18)	0.0991 (18)	0.0007 (12)	−0.0051 (12)	0.0326 (15)
C16	0.0394 (9)	0.0439 (10)	0.0537 (11)	−0.0013 (8)	0.0066 (8)	0.0063 (8)
C17	0.0418 (10)	0.0495 (10)	0.0584 (11)	−0.0075 (8)	−0.0043 (8)	0.0066 (9)
C18	0.0486 (11)	0.0507 (11)	0.0548 (11)	−0.0058 (9)	−0.0048 (8)	0.0122 (9)
C19	0.0400 (10)	0.0462 (10)	0.0547 (11)	−0.0052 (8)	0.0035 (8)	0.0089 (8)
C20	0.0491 (11)	0.0640 (12)	0.0572 (12)	−0.0177 (9)	−0.0084 (9)	0.0136 (10)
C21	0.0528 (12)	0.0614 (12)	0.0524 (11)	−0.0079 (9)	−0.0040 (9)	0.0182 (9)
C22	0.0410 (10)	0.0461 (10)	0.0481 (10)	−0.0039 (8)	0.0035 (8)	0.0052 (8)
C23	0.0498 (11)	0.0492 (11)	0.0635 (12)	−0.0060 (9)	0.0000 (9)	−0.0090 (9)
C24	0.0512 (12)	0.0685 (14)	0.0689 (13)	−0.0140 (10)	−0.0033 (10)	−0.0010 (11)
C25	0.0467 (11)	0.0722 (14)	0.0673 (13)	0.0009 (11)	0.0063 (10)	0.0124 (11)
C26	0.0586 (13)	0.0556 (12)	0.0790 (14)	0.0121 (11)	0.0145 (11)	0.0089 (11)
C27	0.0570 (12)	0.0433 (10)	0.0686 (13)	−0.0050 (9)	0.0059 (10)	0.0018 (9)
C28	0.0526 (15)	0.109 (2)	0.128 (2)	−0.0051 (15)	0.0031 (15)	0.0315 (18)
C29	0.0407 (9)	0.0419 (9)	0.0551 (11)	−0.0065 (8)	−0.0004 (8)	0.0027 (8)
C30	0.0479 (11)	0.0549 (12)	0.0607 (12)	−0.0046 (9)	0.0073 (9)	0.0045 (9)
C31	0.0456 (11)	0.0526 (12)	0.0758 (14)	0.0023 (9)	−0.0004 (10)	−0.0089 (10)
C32	0.0512 (12)	0.0387 (10)	0.0874 (15)	−0.0064 (9)	−0.0086 (10)	0.0035 (10)
C33	0.0591 (13)	0.0557 (12)	0.0847 (15)	−0.0117 (10)	−0.0010 (11)	0.0223 (11)
C34	0.0438 (11)	0.0532 (11)	0.0705 (13)	−0.0042 (9)	0.0064 (9)	0.0122 (10)
C35	0.0667 (16)	0.0538 (14)	0.147 (2)	−0.0014 (12)	−0.0160 (16)	0.0119 (15)
N1	0.0570 (11)	0.0634 (11)	0.0954 (14)	0.0112 (9)	0.0064 (9)	0.0245 (10)
N2	0.0498 (9)	0.0417 (8)	0.0487 (9)	−0.0042 (7)	0.0042 (7)	0.0006 (7)
N3	0.0460 (10)	0.0787 (12)	0.0845 (12)	−0.0026 (9)	−0.0007 (9)	0.0254 (10)
N4	0.0414 (8)	0.0448 (9)	0.0680 (10)	−0.0063 (7)	0.0020 (7)	0.0137 (8)
O1	0.0873 (13)	0.0626 (10)	0.182 (2)	0.0169 (10)	−0.0225 (13)	−0.0063 (11)
O2	0.0574 (12)	0.160 (2)	0.193 (2)	−0.0117 (13)	−0.0086 (13)	0.0392 (18)

### Geometric parameters (Å, º)

**Table:**

C1—N1	1.338 (2)	C18—C19	1.384 (2)
C1—C2	1.385 (3)	C18—H18	0.9300
C1—C6	1.493 (2)	C19—C20	1.381 (2)
C2—C3	1.379 (3)	C19—N4	1.432 (2)
C2—H2	0.9300	C20—C21	1.381 (2)
C3—C4	1.372 (3)	C20—H20	0.9300
C3—H3	0.9300	C21—H21	0.9300
C4—C5	1.362 (3)	C22—C23	1.389 (2)
C4—H4	0.9300	C22—C27	1.391 (2)
C5—N1	1.337 (3)	C22—N4	1.412 (2)
C5—H5	0.9300	C23—C24	1.373 (3)
C6—N2	1.343 (2)	C23—H23	0.9300
C6—C7	1.386 (2)	C24—C25	1.383 (3)
C7—C8	1.389 (2)	C24—H24	0.9300
C7—H7	0.9300	C25—C26	1.378 (3)
C8—C9	1.384 (2)	C25—C28	1.460 (3)
C8—C16	1.486 (2)	C26—C27	1.374 (3)
C9—C10	1.391 (2)	C26—H26	0.9300
C9—H9	0.9300	C27—H27	0.9300
C10—N2	1.350 (2)	C28—O2	1.197 (3)
C10—C11	1.483 (2)	C28—H28	0.9300
C11—N3	1.335 (2)	C29—C34	1.391 (2)
C11—C12	1.383 (2)	C29—C30	1.392 (2)
C12—C13	1.369 (3)	C29—N4	1.411 (2)
C12—H12	0.9300	C30—C31	1.373 (3)
C13—C14	1.366 (3)	C30—H30	0.9300
C13—H13	0.9300	C31—C32	1.388 (3)
C14—C15	1.364 (3)	C31—H31	0.9300
C14—H14	0.9300	C32—C33	1.375 (3)
C15—N3	1.340 (3)	C32—C35	1.461 (3)
C15—H15	0.9300	C33—C34	1.373 (3)
C16—C17	1.388 (2)	C33—H33	0.9300
C16—C21	1.389 (2)	C34—H34	0.9300
C17—C18	1.383 (2)	C35—O1	1.186 (3)
C17—H17	0.9300	C35—H35	0.9300
			
N1—C1—C2	122.04 (18)	C18—C19—N4	121.11 (16)
N1—C1—C6	116.43 (16)	C19—C20—C21	120.15 (17)
C2—C1—C6	121.51 (17)	C19—C20—H20	119.9
C3—C2—C1	119.0 (2)	C21—C20—H20	119.9
C3—C2—H2	120.5	C20—C21—C16	121.59 (17)
C1—C2—H2	120.5	C20—C21—H21	119.2
C4—C3—C2	119.3 (2)	C16—C21—H21	119.2
C4—C3—H3	120.4	C23—C22—C27	118.96 (16)
C2—C3—H3	120.4	C23—C22—N4	121.17 (16)
C5—C4—C3	117.9 (2)	C27—C22—N4	119.85 (16)
C5—C4—H4	121.0	C24—C23—C22	120.17 (18)
C3—C4—H4	121.0	C24—C23—H23	119.9
N1—C5—C4	124.5 (2)	C22—C23—H23	119.9
N1—C5—H5	117.7	C23—C24—C25	121.20 (19)
C4—C5—H5	117.7	C23—C24—H24	119.4
N2—C6—C7	122.61 (16)	C25—C24—H24	119.4
N2—C6—C1	116.73 (15)	C26—C25—C24	118.25 (19)
C7—C6—C1	120.65 (16)	C26—C25—C28	119.3 (2)
C6—C7—C8	120.17 (16)	C24—C25—C28	122.4 (2)
C6—C7—H7	119.9	C27—C26—C25	121.59 (19)
C8—C7—H7	119.9	C27—C26—H26	119.2
C9—C8—C7	117.12 (16)	C25—C26—H26	119.2
C9—C8—C16	121.36 (16)	C26—C27—C22	119.80 (18)
C7—C8—C16	121.43 (15)	C26—C27—H27	120.1
C8—C9—C10	120.08 (16)	C22—C27—H27	120.1
C8—C9—H9	120.0	O2—C28—C25	124.2 (3)
C10—C9—H9	120.0	O2—C28—H28	117.9
N2—C10—C9	122.43 (16)	C25—C28—H28	117.9
N2—C10—C11	116.46 (15)	C34—C29—C30	118.93 (17)
C9—C10—C11	121.10 (16)	C34—C29—N4	120.57 (16)
N3—C11—C12	121.97 (17)	C30—C29—N4	120.49 (16)
N3—C11—C10	116.81 (15)	C31—C30—C29	120.22 (18)
C12—C11—C10	121.22 (16)	C31—C30—H30	119.9
C13—C12—C11	119.52 (19)	C29—C30—H30	119.9
C13—C12—H12	120.2	C30—C31—C32	120.92 (18)
C11—C12—H12	120.2	C30—C31—H31	119.5
C14—C13—C12	119.0 (2)	C32—C31—H31	119.5
C14—C13—H13	120.5	C33—C32—C31	118.40 (18)
C12—C13—H13	120.5	C33—C32—C35	119.8 (2)
C15—C14—C13	118.4 (2)	C31—C32—C35	121.8 (2)
C15—C14—H14	120.8	C34—C33—C32	121.64 (19)
C13—C14—H14	120.8	C34—C33—H33	119.2
N3—C15—C14	124.0 (2)	C32—C33—H33	119.2
N3—C15—H15	118.0	C33—C34—C29	119.87 (18)
C14—C15—H15	118.0	C33—C34—H34	120.1
C17—C16—C21	117.65 (16)	C29—C34—H34	120.1
C17—C16—C8	121.77 (16)	O1—C35—C32	126.6 (3)
C21—C16—C8	120.54 (16)	O1—C35—H35	116.7
C18—C17—C16	121.05 (17)	C32—C35—H35	116.7
C18—C17—H17	119.5	C5—N1—C1	117.21 (19)
C16—C17—H17	119.5	C6—N2—C10	117.55 (14)
C17—C18—C19	120.54 (17)	C11—N3—C15	117.11 (18)
C17—C18—H18	119.7	C29—N4—C22	121.96 (14)
C19—C18—H18	119.7	C29—N4—C19	119.92 (14)
C20—C19—C18	119.02 (16)	C22—N4—C19	118.10 (14)
C20—C19—N4	119.86 (16)		

### Hydrogen-bond geometry (Å, º)

**Table:**

*D*—H···*A*	*D*—H	H···*A*	*D*···*A*	*D*—H···*A*
C27—H27···O1^i^	0.93	2.46	3.339 (3)	158

Symmetry code: (i) −*x*+1/2, *y*+1/2, *z*.
